# Immortalized Rat Tendon-Derived Stem Cells for Tendon Tissue Engineering

**DOI:** 10.3390/bioengineering13030354

**Published:** 2026-03-18

**Authors:** Kat Tik Lau, Hui Wang, Jinxiang Zhang, Dan Michelle Wang, Dai Fei Elmer Ker

**Affiliations:** 1 School of Biomedical Sciences, Faculty of Medicine, The Chinese University of Hong Kong, Hong Kong SAR, China; 1155179685@link.cuhk.edu.hk (K.T.L.); wangmd@cuhk.edu.hk (D.M.W.); 2 Institute for Tissue Engineering and Regenerative Medicine, Chinese University of Hong Kong, Hong Kong SAR, China; 3 Department of Medical Genetics, School of Basic Medicine, Tongji Medical College, Huazhong University of Science and Technology, Wuhan 430030, China; wanghuipitt@hust.edu.cn; 4 Department of Emergency Surgery, Union Hospital, Tongji Medical College, Huazhong University of Science and Technology, Wuhan 430022, China; zhangjinxiang@hust.edu.cn; 5 Department of Orthopaedics and Traumatology, Faculty of Medicine, The Chinese University of Hong Kong, Sha Tin, New Territories, Hong Kong SAR, China; 6 Centre for Neuromuscular Restorative Medicine, Hong Kong Science and Technology Parks, Hong Kong SAR, China; 7 Department of Biomedical Engineering, Faculty of Engineering, The Hong Kong Polytechnic University, Hong Kong SAR, China

**Keywords:** tendon-derived stem cells, immortalized mesenchymal stem cell, tissue engineering

## Abstract

Tendon-derived stem cells (TDSCs) are a unique cell population found in tendons, exhibiting both mesenchymal stem cell (MSC)-like phenotypes and tendon-specific markers. They have emerged as a promising research tool in tendon-related tissue engineering studies. However, there is currently no well-characterized TDSC line with MSC-related phenotypes for investigating tendon biology or developing therapeutics. Here, we established an immortalized monoclonal TDSC, named iTDSC#6, from the Achilles tendon of an adult male Sprague-Dawley rat. Cell clones were characterized for MSC-associated cell surface markers, colony formation capacity, and trilineage differentiation potentials, tenogenic potential and *SV40LT* expression at both early (passage < 10) and late (passage > 30) stages. iTDSC#6 showed stable expression of Simian virus 40 large T antigen (*SV40LT*) and demonstrated similar MSC-like phenotypes as its wild-type counterpart at both early and late passages, including colony formation capability and multi-lineage differentiation potentials. iTDSC#6 was positive for the MSC markers CD90, CD44, CD29 and CD73 (≥95%) and negative for the hematopoietic markers CD34 and CD45 (<1%). Regarding its utility for basic research and therapeutic development, iTDSC#6 showed potential for modelling cells with increased levels of senescence-associated beta-galactosidase activity in response to hydrogen peroxide and for bioengineering scaffold-free, tendon-like 3D constructs as evidenced by its upregulation of tendon-related markers, high nuclear aspect ratio, and aligned collagen organization. In conclusion, an immortalized TDSC line was successfully established that shows promise as a useful research tool to study tendon biology and aid the development of therapeutics for tissue engineering and regenerative medicine.

## 1. Introduction

Tendon injuries are a common musculoskeletal disorder [1,2] and pose significant clinical challenges due to their limited self-healing capacity, which highlight a need for cost-effective treatments that stimulate tissue-resident stem/progenitor cells to enhance regeneration. Presently, the options for clinical treatment and healing outcomes for tendon injuries are limited, which lowers the quality of life and work efficiency of patients [3]. Additionally, the high cost of treatment creates a significant socioeconomic burden on society [4,5]. TDSCs were first identified in mice and human tendons more than two decades ago and are multipotent cells with high expression of both tendon- and mesenchymal stem cell (MSC)-associated markers [6,7], which are critical for tissue homeostasis and injury repair [8,9]. However, they can exhibit impaired regenerative characteristics under pro-inflammatory microenvironments, pathological conditions, and aging. This dysfunction can lead to suboptimal healing processes characterized by the formation of scar tissue and aberrant differentiation into undesirable tissue that contribute towards ectopic bone and cartilage [10,11]. Therefore, further investigations on TDSCs are required for advancing therapeutics for tendon injuries.

Tendon tissue engineering is an actively growing field, driven by the discovery of TDSCs. Indeed, bibliometric analyses have highlighted the importance of TDSCs [12], with a majority of preclinical studies involving rats [9,13] due to their similarities in musculoskeletal structure, tissue composition, and biomechanical attributes compared to humans [14,15], as well as the associated ease of logistics and cost-effectiveness [16]. Studies of TDSCs have advanced basic tendon biology, such as by dissecting subpopulations that contribute to tendon heterotopic ossification [17,18] and revealing signaling pathway(s) that drive tendon fibrosis [19] and degenerative tendinopathy [20]. TDSCs and TDSCs-derived biologics have also demonstrated therapeutic potentials, such as immunomodulation [21], anti-adhesion [22], and accelerating healing [23]. Mechanistic studies revealed that the cellular senescence and aging of TDSCs could be a cause of degenerative tendinopathy [24,25]. However, there are currently no commercially available immortalized TDSC lines, particularly from widely adopted preclinical models such as rats, that are usable as research tools. Existing immortalized cell lines derived from tendon sources often present inadequately characterized and unstable MSC-related phenotypes. On the other hand, TDSCs exhibit considerable heterogeneity with multiple subpopulations, including nestin-positive cells that demonstrate enhanced proliferation rates and greater trilineage differentiation potential [26]. This heterogeneity can vary between passages and among donors, similar to other primary MSCs [27], and with successive passages of primary TDSCs, the expression of stemness markers, MSC-related CD markers, and tendon-related markers tends to decline [28,29]. Therefore, an immortalized monoclonal TDSC line would be an immensely useful tool for researchers.

In this study, we have developed the first immortalized TDSC clone, named iTDSC#6, from the rat Achilles tendon as a novel research tool for advancing basic tendon biology and therapeutic development. iTDSC#6 cells were maintained for more than 30 passages and stably exhibited stem-cell-like characteristics as parental or wild-type TDSCs (WT TDSCs), including high levels of MSC-associated cell surface markers, colony formation capacity, and multi-lineage potential. Furthermore, this cell line can be cultured in conventional serum-containing medium without the need for recombinant growth factor supplementation, resulting in a cost-effective and simple maintenance process. iTDSC#6 displayed similar responses to WT TDSCs in stress-induced senescence and could be bioengineered into 3D tendon-like constructs using scaffold-free approaches. iTDSC#6 presents a promising alternative to WT TDSCs, serving as a standardized cell model for tissue engineering and related research applications ranging from basic to translational studies.

## 2. Materials and Methods

### 2.1. Bibliometric and Commercial Analyses for TDSCs

For academic databases, searching was performed on PubMed, Web of Science and Scopus. For PubMed, the search was performed using various search keys including “immortalized rat tendon-derived stem cell”, “immortalized tendon-derived stem cell”, “immortalized tendon stem cell” and “immortalized tendon cell”. For Web of Science, the search was performed with the search key “(TI = (immortalized) OR TI = (immortalization)) AND (TI = (Tendon-derived stem cells) OR TI = (TDSC) OR TI = (Tendon Stem Progenitor Cell) OR TI = (TSPC) OR TI = (tenocyte))” in Advanced Search. For Scopus, the search was performed using search keys including “TITLE-ABS-KEY (immortalized AND tendon AND cell)” and “TITLE-ABS-KEY (immortalized AND tendon AND stem AND cell)” in Advanced Document Search.

For international cell banks, searching was performed on the official websites of the American Type Culture Collection (ATCC, Manassas, VA, USA, https://www.atcc.org/ (accessed on 13 June 2025)), European Collection of Authenticated Cell Cultures (ECACC, Salisbury, UK, https://www.culturecollections.org.uk/products/cell-cultures/ (accessed on 13 June 2025)) and Japanese Collection of Research Bioresources Cell Bank (JCRB Cell Bank, Osaka, Japan, https://cellbank.nibn.go.jp/english (accessed on 13 June 2025)). Searching on ATCC was performed by inputting the search key “immortalized tendon stem cell” in the search box. Searching on ECACC was performed by clicking on “Browse the entire collection”, and then selecting “Stem cell” and “Stem cell/Fibroblast” on the left panel under “Cell Type”. Searching on JCRB Cell Bank was performed by clicking on “Cell Search” in the top-left corner and then clicking on “Animal/Tissue Order List” under “Cell Line List”. We then used the text search function of the browser to search “tendon” on the web page.

For commercial cell line suppliers, initial searches for immortalized TDSCs were performed with the search engines “Google” and “Bing” using the search key [purchase order “immortalized” “tendon” “stem cell”] (excluding the brackets), and the results were extracted from the first 20 pages. Secondly, after an initial failure to find immortalized TDCSs, a search was performed on the web sites of cell line suppliers identified from these search engines, including AcceGen (Fairfield, NJ, USA, https://www.accegen.com/ (accessed on 13 June 2025)), Applied Biological Materials (Richmond, BC, Canada https://www.abmgood.com/ (accessed on 13 June 2025)), InnoProt (Elexalde Derio, Spain, https://innoprot.com/ (accessed on 13 June 2025)), Cells Online (San Jose, CA, USA, https://cells-online.com/ (accessed on 13 June 2025)) and Creative Bioarray (Shirley, NY, USA, https://www.creative-bioarray.com/ (accessed on 13 June 2025)). Search keys including “immortalized tendon stem cell” and “immortalized stem cell” were used to search for immortalized TDSCs.

### 2.2. Isolation of Primary Rat Tendon-Derived Stem/Progenitor Cells (TDSCs)

All animal experiments were approved by the Animal Experimentation Ethics Committee of the Chinese University of Hong Kong (21-113-MIS). Primary rat TDSCs were isolated according to the “explant” approach from a previous study with minor modifications [30]. In brief, male Sprague-Dawley rats aged about 14 weeks were used for cell isolation. Rats were sacrificed by CO_2_ asphyxia and then the Achilles tendon was excised from both limbs of each rat. An operator, trained and supervised by a senior scientist, performed tendon isolation and dissection. Harvested tendons were transferred into culture dishes and washed with sterile phosphate-buffered saline (PBS) (Gibco, Brooklyn, NY, USA, Cat.: 10010049) washing buffer with antibiotics within a biosafety cabinet and handled aseptically. The paratendon was removed and the surface of the Achilles tendon tissues was gently scraped with a No. 11 surgical scalpel to remove any residual paratendon. Tendon tissues were then minced into about 1 mm^3^ cubic pieces and then immediately transferred and evenly distributed into T-75 flasks and cultured with 13–15 mL of high-glucose Dulbecco’s modified Eagle’s medium (DMEM-HG) (Gibco, Brooklyn, NY, USA, Cat.: 11995073) supplemented with 20% fetal bovine serum (FBS) (Gibco, Brooklyn, NY, USA, Cat.: A5670701) and 1% Penicillin–Streptomycin (P/S) (Gibco, Brooklyn, NY, USA, Cat.: 15140122) at 37 °C in a humidified incubator with 5% CO_2_. Some tendon tissues settled to the bottom of the flask, allowing cells to migrate outward and attach to the culture surface after overnight incubation. Minced tendon tissues were removed after 2 days of incubation during medium renewal. Culture medium was refreshed every 2 to 3 days until the formation of visible cell colonies, which were observed on days 4 to 6. Cells were then trypsinized and mixed, and this initial cell mixture was treated as passage 1 (P1). Cells were then expanded and cell stocks were prepared at P3.

### 2.3. Cell Culture

For routine culture, cells were grown at 37 °C in a humidified incubator with 5% CO_2_ and were seeded at a density of about 3000 cells/cm^2^ with 0.4 mL growth medium (DMEM-HG supplemented with 10% FBS and 1% P/S) per cm^2^ of culture flask. Additionally, 3 μg/mL of puromycin was added into growth medium for culturing immortalized TDSCs. Culture medium was refreshed every 2 days until cells reached 50% to 70% confluence. Cells were then washed once by PBS and then trypsinized by 0.05% trypsin–EDTA (Gibco, Brooklyn, NY, USA, Cat.: 25300062) for 2 min at 37 °C in an incubator with 5% CO_2_. Cells were then detached with gentle tapping and collected by centrifugation at 300 relative centrifugation force (rcf) for 3 min at room temperature. Cells below passage 10 and over passage 30 were considered the early and late passages, respectively. Wild-type TDSCs were used in early passages only while immortalized TDSCs were used in both early and late passages. Microbial contamination was monitored by regular microscopic observation while mycoplasma contamination was checked using a combination of Hoechst 33342 (Thermo Fisher Scientific, Rockford, IL, USA, Cat.: 62249) staining and mycoplasma detection kit (Vazyme, Nanjing, China, Cat.: D201-01). Freezing medium composed of 10% DMSO, 50% FBS, and 40% growth medium was used for all cells, sterile-filtered with a 0.2 μm filter before use. Frozen vials of cells were prepared by resuspending cells with freezing medium pre-cooled on an ice bath. For vials to be thawed in a T-75 flask, 225,000 cells were resuspended per mL of freezing medium for archiving a seeding density of 3000 cells/cm^2^. Freshly prepared vials to be frozen were stored in a Mr. Frosty™ Freezing Container (Thermo Fisher Scientific, Bohemia, NY, USA, Cat.: 5100-0001) and placed in a −80 °C freezer for overnight incubation and then transferred into a liquid nitrogen tank for long-term storage. The cell thawing process was performed by incubating frozen vials in a 37 °C water bath with gentle swirling until there was just a small bit of ice left in the vial; 1 mL of thawed cells was transferred into a 15 mL tube containing 9 mL of growth medium, mixed gently by inverting, and then centrifuged at 300 rcf for 3 min at room temperature. Supernatants were then discarded and the cell pellet was resuspended in 1 mL growth medium by gentle pipetting and used for cell seeding following the routine culture protocol mentioned previously.

### 2.4. Flow Cytometry Analysis

Flow cytometry was performed as previously described [31]. TDCSs were trypsinized, washed once with PBS and then fixed in 4% paraformaldehyde (PFA) (Sigma-Aldrich, St. Louis, MO, USA, Cat.: P6148) for 10 min at room temperature. Cells were then characterized for the expression of the positive MSC-related markers CD29, CD44, CD73, and CD90 and the negative MSC-related markers CD34 and CD45 with single staining with isotype control by a Mesenchymal Stem Cells (Rat) Surface Marker Detection Kit (Cyagen, Jiangsu, China, Cat.: RAXMX-09011) according to the manufacturer’s instructions. Flow cytometry was performed with a FACSymphony A5.2 SORP Flow Cell Analyzer (BD Biosciences, San Jose, CA, USA). A total of 10,000 events were acquired, and forward scatter and side scatter gating were used to exclude debris. A mean fluorescence intensity greater than that of the isotype control was used as the gating threshold for positive staining signals. Data was analyzed by FlowJo (v10) software.

### 2.5. Colony Formation Assay

The colony formation assay was performed as previously described [32] with minor modifications. TDCSs were seeded at a density of 50 cells per well in 6-well plates (SPL Life Sciences, Pocheon-si, Republic of Korea, Cat.: 30006) with 2 mL of growth medium per well. Medium was refreshed every 2 to 3 days for a total culture time of 7 days. Cells were then stained by 1% crystal violet staining solution (Sigma-Aldrich, St. Louis, MO, USA, Cat.: C-3886) and rinsed by deionized water to visualize cell colonies. Samples were then imaged by a digital camera with a white background. Six technical replicates per group were performed.

### 2.6. Trilineage Differentiation

Trilineage differentiation was performed as previously described [33] with minor modifications. Adipogenesis was performed by seeding cells at 4000 cells/cm^2^ in 24-well plates (SPL Life Sciences, Pocheon-si, Republic of Korea, Cat.: 30024) and incubating overnight at 37 °C in an incubator with 5% CO_2_. Undifferentiated cells were treated with growth medium while the differentiation of cells was induced by 50% StemPro Adipogenesis Differentiation medium (Gibco, Brooklyn, NY, USA, Cat.: A1007001) diluted by growth medium. The medium was refreshed every 2 to 3 days for a total culture time of 2 weeks. Cells were then fixed with 4% PFA for 15 min at room temperature and equilibrated with 30% and 60% of isopropanol (RCI Labscan, Bangkok, Thailand, Cat.: AR1162) sequentially. Samples were then stained by 0.3% Oil Red O (ChemCruz, Dallas, TX, USA, Cat.: sc-203749) in 60% isopropanol for 15 min at room temperature and then washed 3 times with 60% isopropanol and once with deionized water. Three technical replicates per group were performed.

Chondrogenesis was performed as previously described [34,35,36] with minor modifications. Cells were seeded at 450,000 cells/cm^2^ in 96-well plates (SPL Life Sciences, Pocheon-si, Republic of Korea, Cat.: 30096) by centrifugation at 500 rcf for 5 min and then the medium was immediately replaced by either StemPro Chondrogenesis Differentiation medium (Gibco, Brooklyn, NY, USA, Cat.: A1007101) or growth medium in the differentiation and control groups, respectively. Cells were incubated at 37 °C in an incubator with 5% CO_2_ and the medium was refreshed every 2 to 3 days for a total culture time of 2 weeks. Cells were then fixed with 4% PFA for 15 min at room temperature and washed 3 times with PBS. Samples were stained with 1% Alcian blue staining solution (Electron Microscopy Sciences, Morgantown, PA, USA, Cat.:26116-06) for 10 min and then washed 3 times using deionized water. Three technical replicates per group were performed.

Osteogenesis was performed by seeding cells at 4000 cells/cm^2^ in 24-well plates (SPL Life Sciences, Pocheon-si, Republic of Korea, Cat.: 30024) and incubating overnight at 37 °C in an incubator with 5% CO_2_. Undifferentiated cells were treated with growth medium while the differentiation of cells was induced by osteogenic medium composed of 50 μg/mL ascorbic acid (Sigma-Aldrich, St. Louis, MO, USA, Cat.: A5960), 10 mM β-glycerolphosphate (Sigma-Aldrich, St. Louis, MO, USA, Cat.: G9422) and 100 ng/mL BMP-2 (Sino Biological, Beijing, China, Cat.: 10426-HNAE-20) in the growth medium. The medium was refreshed every 2 to 3 days for a total culture time of 4 weeks. Cells were then washed with PBS and fixed with 10% formalin (Epredia, Kalamazoo, MI, USA, Cat.: 5735SSC) for 15 min followed by 3 washes with deionized water. Cells were then stained with 2% Alizarin Red S (Sigma-Aldrich, St. Louis, MO, USA, Cat.: A5533) solution at pH 4.1 for 1 h and washed 5 times with deionized water.

Samples were then imaged by a Primovert inverted cell culture microscope (ZEISS, Jena, Germany) with an Axiocam 208 color camera to identify red-colored oil droplet-positive cells, blue-colored spheroids, and red-colored calcium deposit nodules for confirming adipogenesis, chondrogenesis and osteogenesis, respectively. Three technical replicates per group were performed.

### 2.7. Generation of Monoclonal Immortalized TDSCs

Monoclonal iTDSC#6 was generated by seeding WT TDSCs at 5000 cells/cm^2^ in 24-well plates, incubating overnight and then transducing with self-inactivating SV40 large T antigen (SV40LT) lentiviral particles (GeneCopoeia, Rockville, MD, USA, Cat.: LP742-025) at a multiplicity of infection of 5 to 10 with 5 μg/mL of polybrene (Sigma-Aldrich, St. Louis, MO, USA, Cat.: TR-1003) in growth medium for 24 h. Cells were then washed with PBS and then incubated in blank growth medium for 48 h. Selections of positively transduced cells were then performed by culturing cells with 5 μg/mL of puromycin (Sigma-Aldrich, St. Louis, MO, USA, Cat.: P9620), which is the optimal concentration for killing WT TDSCs (Figure S2). Growth medium with 5 μg/mL of puromycin was refreshed every 48 h until the formation of visible cell colonies. Cells were then trypsinized and replated into a culture flask with 5 μg/mL of puromycin for expansion of positively transduced cells. Random single-cell clones of iTDSCs were directly sorted into 96-well plates by single-cell sorting using a FACSAria Fusion Cell sorter and Cell Analyzer (BD Biosciences, San Jose, CA, USA) and expanded in growth medium with 3 μg/mL of puromycin. Several cell clones were expanded, including iTDSC#6, with a high proliferation rate and wild-type-like elongated cell morphology. Slow proliferative clones with flattened, enlarged cell morphology were excluded as these features are associated with cellular senescence [37]. A total of 6 clones were frozen and three of them, including iTDSC#6, were selected for initial cell characterization experiments. These clones were cultured in the same way as WT TDSCs. Cells were then expanded and cell stocks were prepared at P3. Characterization assays for MSC-related phenotypes including flow cytometry analysis, colony formation assay and trilineage differentiation were performed on cells at early (less than 10) and late passages (greater than 30) using the same protocols for WT TDSCs previously mentioned.

### 2.8. Determining Puromycin Resistance in WT TDSCs

WT TDSCs were seeded at 5000 cells/cm^2^ in 96-well plates and incubated overnight at 37 °C in an incubator with 5% CO_2_. Culture medium was then replaced with growth medium containing 0, 0.5, 1, 2, 3, 4, 5, 6, 7 and 8 μg/mL of puromycin and incubated for 48 h to determine the optimal drug concentration for suppressing cell proliferation and inducing cell death. Samples were observed and recorded using a Primovert inverted cell culture microscope (ZEISS, Jena, Germany) with Axiocam 208 color camera with bright-field imaging. Three technical replicates per group were performed.

### 2.9. Gene Expression Analysis

Quantitative real-time polymerase chain reaction (qPCR) was performed as previously described [38] with minor modifications. To verify gene expression of *SV40LT* in immortalized TDSCs, total RNA was extracted from WT TDSCs at early passage and iTDSC#6 at early and late passage by RNAiso Plus (Takara Bio, Shiga, Japan, Cat.: 9109) according to the manufacturer’s instructions. RNA concentration and quality were determined by a NanoDrop spectrophotometer (Thermo Fisher Scientific, Bohemia, NY, USA). cDNA was synthesized by reverse transcription of 1 μg total RNA using LunaScript RT Supermix (New England Biolabs, Ipswich, MA, USA, Cat.: E3010L). qPCR was performed using a QuantStudio 7 (Flex) Real-time PCR System (Applied Biosystems, Waltham, MA, USA) with 2X Luna Universal qPCR Master Mix (New England Biolabs, MA, USA, Cat: M3003S). The primers used were: *Gapdh* (5′-TGTACCACCAACTGCTTAGC-3′ + 5′-GGCATGGACTGTGGTCATGAG-3′) and *SV40LT* (5′-AAGTTTAATGTGGCTATGGG-3′ + 5′-ACTGTGAATCAATGCCTGTT-3′). The PCR cycling conditions were as follows: an initial denaturation at 95 °C for 1 minute, followed by 40 cycles of denaturation at 95 °C for 15 seconds and extension at 60 °C for 30 s, ending with a 60 to 95 °C melt curve. The results were shown as relative expression to the housekeeping *Gapdh* gene using the formula 2^−∆CT^. Three technical replicates per group were performed.

### 2.10. Senescence-Associated Beta-Galactosidase Activity Staining Assay

A beta-galactosidase activity staining assay was performed to evaluate the basal cellular senescence status and oxidative stress-induced cellular senescence responses in TDSCs. Cells were seeded at 1500 cells/cm^2^ and incubated overnight at 37 °C in a humidified incubator with 5% CO_2_ and then treated with growth medium or 400 μM of H_2_O_2_ (VWR, Wayne, PA, USA, Cat.: 23615.261) for 2 h in the control or oxidative stress-induced groups, respectively. Cells were then washed once with PBS and replaced with normal growth medium and incubated for 48 h. Cells were then fixed with 4% PFA for 10 min and processed using a senescence-associated beta-galactosidase staining kit (Beyotime, Shanghai, China, Cat.: C0602) according to the manufacturer’s instructions. Cells were then washed with PBS and nuclei were stained by Hoechst 33342 (4 μM, Thermo Fisher Scientific, Bohemia, NY, USA, Cat.: 62249). Blue-colored beta-galactosidase-positive cells and nuclei were imaged by an ECLIPSE Ti2-A (Nikon, Kanagawa, Japan) microscope in bright-field and fluorescent modes, respectively. No filter was used when capturing bright-field images while a Multi LED set multi-band excitation/multi-band emission filter set (CHROMA, Bellows Falls, VT, USA, Cat.: 89402) was used with DAPI-equivalent excitation wavelength for capturing fluorescent images of nuclei. Images of nuclei were processed by ImageJ software (1.53v) with the StarDist 2D plugin (0.3.0) to estimate cell density. For images where it was qualitatively determined that StarDist 2D did not segment images well, the number of beta-galactosidase-positive cells were manually labelled and then divided by total nucleus number to generate the percentage of cells positive for beta-galactosidase activity. Three biological replicates per group were performed.

### 2.11. Modification of 6-Well Plate for Scaffold-Free 3D Tenogenesis

To make cylindrical anchors, one end of a P-1000 pipette tip rack was taped to serve as a mold to make cylindrical polydimethylsiloxane (PDMS) (Dow, Midland, MI, USA, SYLGARD 184 Silicone Elastomer Kit) according to the manufacturer’s instructions. After demolding, PDMS anchors were sterilized by 70% ethanol wash, air-dried and UV-illuminated in a biosafety cabinet for 30 min. Two PDMS anchors placed 5 mm apart were glued onto each well of a 6-well plate using sterile high-vacuum grease (Dow Corning, Midland, MI, USA) following the middle line of the well.

### 2.12. Tenogenesis Assay

Tenogenesis assays were performed as previously described [39] with minor modifications. TDSCs were differentiated in a 2D monolayer for early (4 days) and late (7 days) time points. Cells were seeded at densities of 10,000 cells/cm^2^ and 1500 cells/cm^2^ in 24-well plates for the early and late time points, respectively, and incubated overnight at 37 °C in an incubator with 5% CO_2_. Tenogenesis was induced by tenogenic medium composed of 50 ug/mL ascorbic acid, 50 ng/mL FGF-2 (PeproTech, Cranbury, NJ, USA, Cat.: 100-18B) and 10 ng/mL TGF-β3 (ProspecBio, Ness Ziona, Israel, Cat.: CYT-368) in growth medium. The control group was treated with growth medium, which was refreshed every 2 to 3 days. Cells were harvested after days 4 or 7 of culture and fixed with 4% PFA for 15 min at room temperature. Three biological replicates per group were performed.

TDSCs were differentiated using a scaffold-free 3D model for the early (9 days) and late (21 days) time points. Cells were seeded at a density of 10,000 cells/cm^2^ in normal or modified 6-well plates for the control and 3D differentiation groups, respectively, and incubated overnight at 37 °C in an incubator with 5% CO_2_. Tenogenesis was induced by tenogenic medium and the control group was treated with growth medium. The medium was refreshed every 2 to 3 days. Cells or 3D constructs were harvested after day 9 or 21 of culture and fixed with 4% PFA for 60 min at room temperature. 3D constructs were then washed with PBS and equilibrated with 30% sucrose solution prior to embedding in Tissue-Tek O.C.T. Compound (Sakura Finetek, Torrance, CA, USA, Cat.: 4583) for cryo-sectioning. 3D constructs were sectioned with 10 μm thickness for immunofluorescence and cytochemical staining assays. Three biological replicates per group were performed.

### 2.13. Picrosirius Red Staining Assay

Cryosections were stained with 1% picrosirius red staining solution (Sigma-Aldrich, St. Louis, MO, USA, Cat.: 365548) for 1 h and washed 3 times with 0.5% acetic acid (VWR, Wayne, PA, USA, Cat.: 20104.334) in water. Samples were dried and imaged using an ECLIPSE Ni-U (Nikon, Kanagawa, Japan) microscope with phase contrast and polarized light imaging.

### 2.14. Immunofluorescence Staining

Immunofluorescence staining was performed as previously described [32,39,40,41,42,43,44,45] with minor modifications. For 2D and 3D tenogenesis assays, scleraxis (SCX) staining was performed on samples with permeabilization by 0.2% Triton X-100 (Sigma-Aldrich, St. Louis, MO, USA, Cat.: X100) for 20 min at room temperature, while tenomodulin (TNMD) and type I collagen (COL1) were stained without permeabilization. Samples were blocked by 10% donkey serum for 20 min at room temperature prior to probing with the following primary antibodies overnight at 4 °C: mouse anti-SCX (2 μg/mL, Santa Cruz, CA, USA, Cat.: sc-518082), rabbit anti-TNMD (1 μg/mL, Abcam, Cambridge, UK, Cat.: ab203676) and goat anti-COL1 (0.8 μg/mL, SouthernBiotech, Birmingham, AL, USA, Cat.: 1310-01). Samples were then washed with 0.1% Tween 20 (Sigma-Aldrich, St. Louis, MO, USA, Cat.: P9416) in PBS and probed with the following secondary antibodies: donkey anti-mouse Alexa Fluor 647 (2 μg/mL, Invitrogen, Carlsbad, CA, USA, Cat.: A-31571), donkey anti-goat Alexa Fluor 555 (2 μg/mL, Invitrogen, Carlsbad, CA, USA, Cat.: A-21432) and donkey anti-rabbit Alexa Fluor 647 (1 μg/mL, Invitrogen, Carlsbad, CA, USA, Cat.: A-31573). Nuclei were stained by Hoechst 33342 (4 μM).

For detecting the basal protein expression of tendon-related markers in WT TDSCs and iTDSC#6, cells were seeded at a density of 5000 cells/cm^2^ and incubated overnight. Samples were then fixed with 4% PFA and permeabilized by 0.2% Triton X-100 for staining SCX, TNMD and COL1 using the same concentration of primary and secondary antibodies as mentioned before.

All samples were imaged with an ECLIPSE Ti2-A fluorescent microscope. A Multi LED set multi-band excitation/multi-band emission filter set was used with DAPI-, TRITC-, and Cy5-equivalent excitation wavelengths for capturing fluorescent signals for Hoechst 33342, Alexa Fluor 555 and Alexa Fluor 647, respectively. Images were then processed by ImageJ software (1.53v) for measuring mean pixel intensity. Cell density was estimated by nucleus counting, with the nuclei of 2D samples grown on tissue culture plates processed by the StarDist 2D plugin (0.3.0), while the nuclei of sectioned 3D samples were manually counted. Manual counting was chosen as it was qualitatively determined that StarDist 2D did not segment images from 3D samples well. Semiquantification was performed by dividing mean pixel intensity by nucleus number to generate the cell density-normalized fluorescence signal.

### 2.15. Statistical Analysis

Statistical analysis was conducted using SPSS Statistics 24 (IBM, Armonk, NY, USA). Normality and equal variances among groups were tested by the Shapiro–Wilk test and the Levene test, respectively. Visual inspections of QQ plots were performed to ensure the validity of normality assumptions such as absence of outliers and obvious skews in distribution shape. Three independent biological experiments were utilized as the unit of analysis with averaging of technical replicates to generate values for each independent data point for statistical analysis. Two-tailed unpaired *t*-tests or one-way ANOVAs were performed as indicated in figure legends. An unpaired *t*-test without or with Welch’s correction was performed to determine statistical significance between two groups with or without equal variance, respectively. One-way ANOVA was performed using a post hoc test using Tukey’s honestly significant difference test or Dunnett’s T3 test for samples with or without equal variance, respectively.

## 3. Results

### 3.1. Limited Availability of Immortalized TDSCs

To assess the need for an immortalized cell line for TDSCs, a bibliometric research and commercial product analysis was performed. For bibliometric research analysis, the search was performed in academic databases including PubMed, Web of Science, and Scopus, which retrieved two immortalized cell lines of tendon origin: TT-D6 [46] and immortalized murine tenocytes (IMTs) [47]. Notably, both cell types are typically regarded as tissue-resident fibroblasts/tenocytes as opposed to stem/progenitor cells in the literature [48,49]. For commercial product analysis, the search was performed in cell banks including the American Type Culture Collection (ATCC), European Collection of Authenticated Cell Cultures (ECACC) and Japanese Collection of Research Bioresources Cell Bank (JCRB Cell Bank) and on the internet. These searches did not yield any tendon-related cell lines other than immortalized tenocytes (Figure S1). Overall, no published academic reports or commercially available sources of well-characterized TDSCs with long-term MSC-related phenotypes were identified.

### 3.2. SV40LT-Immortalized TDSC#6 Maintained Wild-Type-Like MSC-Associated Phenotypes

**Figure 1:**
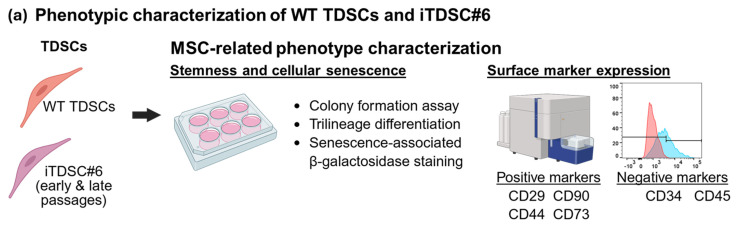

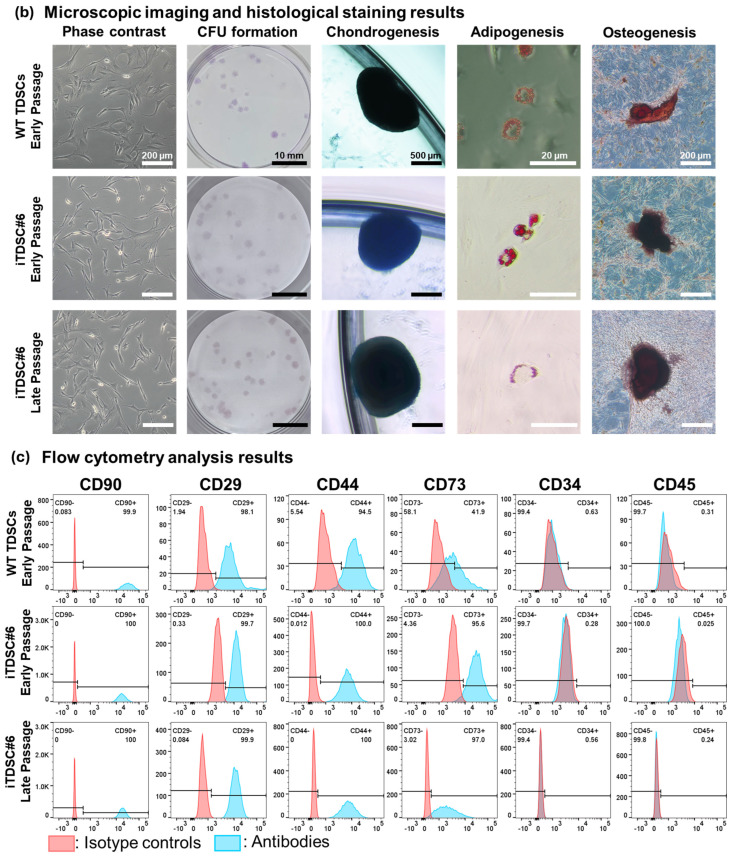
MSC-related phenotype characterizations of WT TDSCs at early passages, as well as iTDSC#6 at early and late passages. (**a**) Overview of assays performed to characterize WT TDSCs and iTDSC#6. (**b**) Phase contrast images showed that WT TDSCs and iTDSC#6 had similar cell morphology and positive staining for crystal violet, Alcian blue, Oil Red O, and Alizarin Red S at early and late passages, which indicated capability for colony formation and trilineage differentiation, respectively. Scale bars as indicated. (**c**) Flow cytometry analysis was performed on WT, early-passage iTDSC#6 and late-passage iTDSC#6 at passages 5, 3 and 33, respectively. All groups exhibited high proportions (about 95%) of MSC-related markers like CD90, CD29 and CD44 and low proportions (below 1%) of hematopoietic-related markers like CD34 and CD45. Interestingly, iTDSC#6 expressed the MSC-associated CD73 marker at a relatively high proportion (more than 95%) compared to WT TDSCs (about 40%).

To investigate the pro-regenerative attributes of iTDSCs, the MSC-associated phenotypes of WT TDSCs and a SV40LT-immortalized clone termed iTDSC#6 were characterized at early (<10) and late (>30) passages via colony formation, multi-lineage differentiation, and flow cytometry assays (Figure 1a). The optimal puromycin concentration for *SV40LT*-transduced clone selection was found to be 5 μg/mL (Figure S2). *SV40LT* gene expression in iTDSC#6 was verified by qPCR (Figure S3). WT TDSCs isolated from rat Achilles tendons exhibited MSC-related phenotypes capable of colony formation and trilineage differentiation (Figure 1b,c), with a high proportion of cells expressing positive MSC markers like CD90 (99.9%), CD29 (98.1%) and CD44 (94.5%). Another MSC-associated marker, CD73, was also expressed in about 42% of cells (Figure 1c). iTDSC#6 highly resembled WT TDSCs in terms of MSC-related phenotypes and cell morphology regardless of early or late passages. Interestingly, iTDSC#6 showed a higher proportion of cells positive for the MSC-related marker CD73 (≥95%) compared to WT TDSCs (42%) (Figure 1c). Additionally, the tendon origin of these cells was confirmed by basal expression of tendon-related markers such as scleraxis (SCX), tenomodulin (TNMD), and type I collagen (COL1) for both WT TDSCs and iTDSC#6 at both early and late passages (Figure S4). Overall, iTDSC#6 cells exhibited similar stem cell-like phenotypes to their WT TDSC counterparts.

### 3.3. iTDSC#6 Showed Tenogenesis Capability

**Figure 2:**
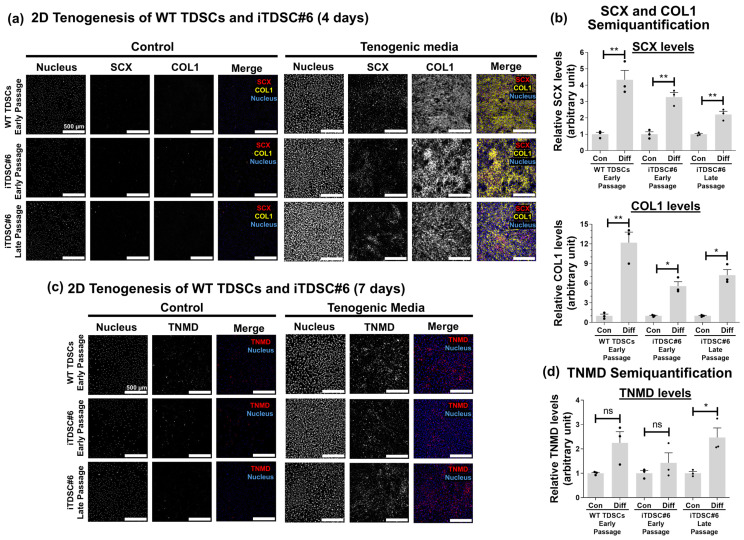
2D tenogenesis of WT TDSCs and iTDSC#6 in tenogenic media on plain tissue culture polystyrene (TCP) at early (4 days) and later (7 days) time points. (**a**) At day 4, immunofluorescence (IF) staining showed that the tenogenic marker SCX and tendon ECM marker COL1 were upregulated in the tenogenic media group compared to the control group. (**b**) Semiquantification of SCX and COL1. (**c**) At day 7, immunofluorescence staining showed that TNMD levels were upregulated in the tenogenic media group compared to the control group. (**d**) Semiquantification of IF staining for TNMD. Bar charts are shown as mean ± SEM, two-tailed unpaired *t*-test, and with Welch’s correction where applicable, *n* = 3 independent experiments, each with 3 technical replicates to assess inter- and intra-experimental variability. ns: *p* > 0.05, *: *p* ≤ 0.05, **: *p* ≤ 0.01. Scale bar indicates 500 μm.

A key defining feature of TDSCs is their ability to undergo tendon differentiation [29,50]. To investigate if iTDSC#6 preserved tenogenic potential after SV40LT immortalization, 2D tenogenesis of iTDSC#6 was performed and the expression of tenogenic markers like SCX and TNMD, as well as the tendon extracellular matrix (ECM)-related marker COL1, was evaluated by immunofluorescence staining. Both WT TDSCs and iTDSC#6 at early and late passages were evaluated by culturing with tenogenic media composed of 10 ng/mL TGF-β3, 50 ng/mL FGF-2, and 50 μg/mL ascorbic acid, and assessed at early (4 days) and late (7 days) differentiation time points. At day 4, SCX transcription factor levels were increased about 2- to 4-fold and mostly detected in the nuclear region in the treatment groups but not in the control groups. Additionally, COL1 was stained extracellularly with a network-like morphology in the treatment groups but not in the control groups (Figure 2). At day 7, extracellular TNMD was also increased by about 2.5-fold in iTDSC#6 at later passages. No upregulation of TNMD was observed for WT TDSCs and iTDSC#6 at early passages. Overall, these results demonstrated that iTDSC#6 cells were responsive to established tenogenic-promoting factors and underwent tenogenesis similarly to their WT TDSC counterparts.

### 3.4. iTDSC#6 Exhibited a Low Level of Cellular Senescence After Repeated Passage Resembling WT TDSCs at Early Passage

**Figure 3:**
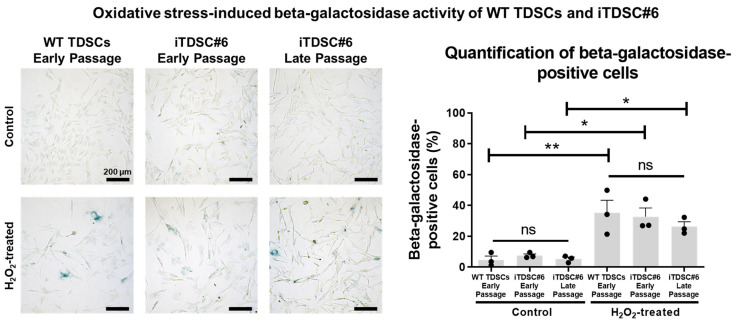
Beta-galactosidase activity staining of WT TDSCs at early passages as well as iTDSC#6 at early and late passages under normal culture conditions and under H_2_O_2_-induced oxidative stress. WT, early-passage iTDSC#6 and late-passage iTDSC#6 showed low levels of cellular senescence, as indicated by less than 8% of cells expressing beta-galactosidase activity. Oxidative stress-induced cellular senescence using 400 μM of H_2_O_2_ treatment resulted in about 30% of cells expressing beta-galactosidase. One-way ANOVA with Tukey post-test was performed. No significant differences were observed among three cells within the control or H_2_O_2_ treatment groups. *n* = 3 independent experiments, each with 3 technical replicates to assess inter- and intra-experimental variability. ns: *p* > 0.05, *: *p* ≤ 0.05, **: *p* ≤ 0.01. Scale bar indicates 200 μm.

Hydrogen peroxide (H_2_O_2_) is a useful inducer of oxidative stress for studying and modelling aging-induced senescence [51]. To determine if TDSC#6 can be useful as a cell model for oxidative stress-induced senescence, a senescence-associated beta-galactosidase activity assay was performed on cells treated with and without H_2_O_2_. iTDSC#6 at both early and late passages exhibited a low percentage of beta-galactosidase-positive cells, similar to WT TDSCs at early passage (≤8%). All cells were sensitive to H_2_O_2_ treatment and exhibited about 3-fold increased beta-galactosidase activity relative to non-treated control groups (Figure 3). Overall, iTDSC#6 cells exhibited low levels of cellular senescence and exhibited hydrogen peroxide-induced senescence-like phenotypes similar to their WT TDSC counterparts.

### 3.5. Bioengineered, Scaffold-Free 3D Tendon-like Constructs Using iTDSC#6

3D tenogenesis can better mimic the native tendon microenvironment and promote formation of a more matured tenocyte-like phenotype compared to 2D culture alone [52]. To investigate if TDSC#6 could undergo scaffold-free 3D tenogenesis, the cells were cultured under high density in the presence of tenogenic media and two PDMS posts for facilitating the formation and contraction of a cell sheet to generate a 3D tendon-like construct (Figure S5). Macroscopic observations showed that TDSCs formed 3D constructs with a rubber band-like appearance in the presence of PDMS anchors and a rolled-up cell sheet in their absence (Figure S5).

**Figure 4:**
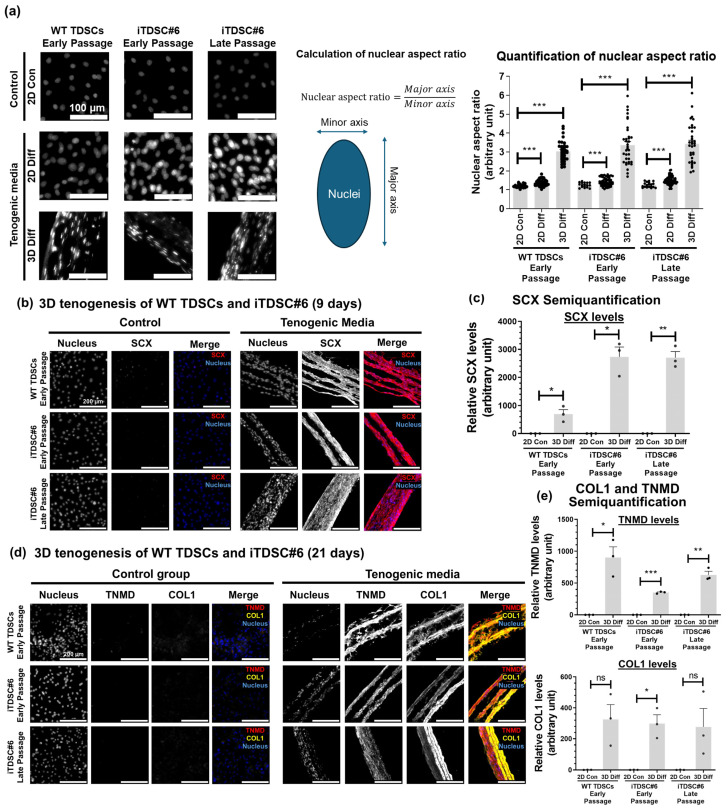
Novel scaffold-free 3D tenogenesis model of TDSCs. (**a**) Scaffold-free 3D tenogenesis after 21 days of culture resulted in cells with high nuclear aspect ratios (NARs) ranging from 2 to 6, while cells in 2D culture exhibited NARs ranging from 1.5 to 1.8. Bar charts are shown as mean ± SEM. Approximately 20 to 70 nuclei were randomly chosen and measured for each group. One-way Welch’s ANOVA test with Dunnett’s T3 post-test was performed. ***: *p* ≤ 0.001. Scale bar indicates 100 μm. (**b**) IF staining of microtissue cultured for 9 days for the early tenogenic marker SCX. Strong SCX signals were detected in 3D constructs in the differentiation group, while very low SCX signals were detected in the control group. (**c**) Semiquantification of SCX levels. (**d**) IF staining of microtissue cultured for 21 days for the tenocyte maturation marker TNMD and the ECM marker COL1. The 3D construct showed an aligned fibrous-like structure with highly expressed TNMD and COL1 in the differentiation group. Minimal signals were detected in the control group. (**e**) Semiquantification of TNMD and COL1 levels. Bar chart is shown as mean ± SEM, two-tailed unpaired *t*-test, and with Welch’s correction where applicable, *n* = 3 independent experiments, each with 3 technical replicates to assess inter- and intra-experimental variability. ns: *p* > 0.05, *: *p* ≤ 0.05, **: *p* ≤ 0.01, ***: *p* ≤ 0.001. Scale bar indicates 500 μm.

To assess if the 3D construct possesses tendon-like characteristics, the nuclear morphology and tendon marker levels, as well as the ECM alignment, of WT TDSCs and iTDSC#6 at early and late passage were analyzed at 9- and 21-days post-seeding. At day 21, cells within 3D constructs exhibited higher nuclear aspect ratio (NAR) values ranging from 2 to 6 compared to undifferentiated or tenogenically differentiated cells in 2D, with NAR values ranging from 1.5 to 1.8 (Figure 4a). Also, immunofluorescence staining of 3D microtissue for tendon-related markers like SCX, TNMD, and COL1 was performed (Figure 4b–e). At day 9, SCX levels were increased by about 700- and 2700-fold in WT TDSCs and iTDSC#6 at early and late passages, respectively, relative to their respective undifferentiated control group (Figure 4c). Minimal signals of the tendon-related markers SCX, TNMD, and COL1 were detected in undifferentiated WT TDSCs and iTDSC#6 at both day 9 and 21 (Figure 4b,d). At day 21, TNMD levels were upregulated by about 900-, 350-, and 630-fold in WT TDSCs, iTDSC#6 at early passage, and iTDSC#6 at late passage, respectively, relative to their respective undifferentiated control groups (Figure 4e). Moreover, COL1 levels were upregulated 300-fold in iTDSC#6 at early passage relative to the undifferentiated control group (Figure 4e). Additionally, picrosirius red staining of 3D differentiated samples revealed native tendon-like strong birefringence signals, indicating aligned collagen molecules (Figure S6). Overall, iTDSC#6 cells formed engineered scaffold-free, tendon-like tissue constructs and exhibited elongated nuclear morphology, increased levels of tendon-associated markers, and aligned ECM deposition similar to their WT TDSC counterparts.

## 4. Discussion

In this study, we successfully established the first immortalized rat TDSC line, designated iTDSC#6, which exhibits long-term, stem cell-like characteristics. Various critical attributes of iTDSC#6, including immortalization gene expression, low levels of cellular senescence, MSC-related surface marker expression, colony formation capacity and trilineage differentiation potentials, were sustained after long-term culture, thus addressing a critical technical gap in the availability of such research tools in the field. iTDSC#6 complements existing immortalized cell lines derived from tendons (Figure S1), such as TT-D6 and IMT, which are typically regarded as tendon-resident fibroblasts/tenocytes [46,47]. Our studies showed that iTDSC#6 retained MSC-like phenotypes at both early and late passages, including CD marker expression, colony formation capacity, and trilineage differentiation potential, alongside basal levels of tendon-related markers and tenogenic potential over at least 30 passages. As a monoclonal cell line with reduced intrinsic heterogeneity, iTDSC#6 offers significant advantages over cells with polyclonal subpopulations, which often exhibit increased variability due to donor differences, batch effects, or passage number, thereby positioning iTDSC#6 as a pivotal tool for advancing both academic research and industrial drug development related to tendon studies.

Immortalized TDSCs were generated from rats to provide a valuable resource for preclinical investigations. Specifically, rats are one of the most commonly used species in tendon injury and regeneration studies [53] because of the high similarity between rat and human rotator cuffs [14] and Achilles tendons [15] in terms of musculoskeletal anatomical structure, biomechanical characteristics, and tissue composition. As an animal model, conducting experiments on rats is affordable and logistically easier to manage compared to larger animal models such as rabbits or goats. At the same time, rats are large enough to allow for surgical manipulation that resembles clinically used procedures compared to smaller animal models such as mice [16]. This cell line was immortalized using the SV40LT antigen, which is a widely used immortalization agent [54] that has successfully immortalized MSCs with diverse tissue and organ origins [55,56,57,58,59]. Studies reported that the expression of MSC surface markers like CD73 may decrease after *SV40LT* transduction in various MSCs [56,60], which appears contrary to our current finding of increased CD73 in iTDSC#6. However, we attribute the elevated CD73 expression in iTDSC#6 mainly to clone-specific variation rather than a general effect of SV40LT. During cell clone selection, we prioritized clones with high proliferation rates and spindle-shaped morphology, which are the typical characteristics of non-senescent MSCs [37,61,62] that generally show higher MSC-related surface markers, including CD73 [63]. Thus, the increased CD73 expression in iTDSC#6 likely reflects successful selection of a non-senescent, MSC-like clone. More importantly, iTDSC#6 retains the wild-type-like MSC and tendon-related phenotypes, including the expression of MSC CD markers like CD29, CD44, CD90, and CD73, colony formation capacity, trilineage differentiation potentials, and tendon markers such as SCX, TNMD, and COL1. Both wild-type and immortalized TDSCs exhibited similar tenogenic potential and responses towards a tenogenic cocktail comprising TGF-β3, FGF-2, and ascorbic acid [43], resulting in the upregulation of tendon-related markers and ECM synthesis. On one hand, iTDSC#6 as a monoclonal cell line could improve consistency and reproducibility in experimental results, as the cell source is a major cause of experimental variability [64]; additionally, cell immortalization allowed enhanced cell expansion capacity, providing a virtually unlimited supply of cells and saving resources spent on isolating primary TDSCs. Therefore, iTDSC#6 can be used as a widely accessible useful research tool that can potentially substitute for wild-type TDSCs in tendon-related studies.

As a research-only tool, iTDSC#6 has potential use for studying basic biology and exploring potential translational research applications. In basic research, iTDSC#6 exhibits low levels of basal cellular senescence and responsiveness to oxidative stress-induced senescence, making it a potential model for studying senescence-related degenerative tendinopathy [20]. For translational research, a scaffold-free 3D tenogenesis model utilizing iTDSC#6 was demonstrated, enabling the generation of 3D tendon-like microtissues with native-like nuclear morphology and expression of tendon markers. This scaffold-free approach is simplistic and does not require complex bio-fabrication approaches such as 3D printing [65] or tedious manual manipulation of cell sheets [66]. Indeed, the NAR values of cells from the current 3D model is comparable to other sophisticated models [67,68] and native tendons [69]. This simple 3D tenogenesis model holds promise for studying TDSC ECM synthesis and turnover, and could be integrated into microfluidic devices as “tendon-on-a-chip” systems [70]. Additionally, therapeutic effects of TDSC-derived exosomes [71] and tendon ECMs [42] have been demonstrated by previous studies, and similar materials could be generated by iTDSC#6 cells in the 2D or 3D tenogenesis models. Additionally, modifications like gene editing of the cell line and decellularization could be applied to improve the therapeutic potency of exosomes [72] and reduce the immunogenicity of ECMs [73], respectively. Finally, iTDSC#6 showed a similar response to WT TDSCs toward tenogenic factors, and could therefore be used as an alternative cell line for drug screening assays. Overall, iTDSC#6 could potentially support fundamental research with a wide range of applications in tissue engineering and regenerative medicine.

In addition, further studies will be needed to determine if iTDSC#6 exhibits characteristics representative of the broad range of subpopulations reported for TDSCs, since single-cell RNA-seq studies have shown that there are multiple stem/progenitor subtypes in the tendon [26,74,75]. Future studies may be employed to assess if iTDSC#6 exhibits a similar gene expression profile as their wild-type counterparts. Global transcriptomic analysis would provide deeper insights into the effects of SV40LT on the overall gene expression profile of TDSCs. Furthermore, characterization of additional clones may enable the identification of TDSC clones exhibiting distinct phenotypes and functional properties that may allow for studies of TDSC heterogeneity. Alternatively, immortalized TDSC sub-clones with different properties and functionalities may be established in order to address the specific limitations of iTDSC#6. Future therapeutic or industrial applications of products derived from this cell line, such as secreted factors, extracellular vesicles, and extracellular matrices, will also require evaluations of their long-term safety and efficacy.

## 5. Conclusions

We have successfully generated iTDSC#6, a monoclonal SV40LT-immortalized rat TDSC line. iTDSC#6 exhibits long-term MSC-associated phenotypes including stem-cell-associated attributes, stress-induced senescence, and the ability to undergo scaffold-free 3D tenogenesis. iTDSC#6 has the potential for a wide range of basic and translational applications, including studies of tendon biology, as well as tissue engineering and regenerative medicine.

## Data Availability

All data supporting the findings of this study are available within the paper and its Supplementary Information. Raw data will be made available upon reasonable request.

## Supplementary Materials

The following supporting information can be downloaded at https://www.mdpi.com/article/10.3390/bioengineering13030354/s1, Figure S1: PRISMA flow diagrams of immortalized tendon-derived stem cells in academic databases, non-profit cell banks and commercial cell line suppliers. Bibliometric analysis and commodity search retrieved two immortalized cell lines displaying MSC-like phenotypes with tendon origin, including immortalized murine tenocyte and TT-D6 cells; however, no rat-tendon-derived stem cell lines were found; Figure S2: Puromycin resistance of WT TDSCs was determined by visual inspection of cell growth. Cells were cultured in growth medium containing up to 8 μg/mL of puromycin for 48 h in 96-well plates. The optimal killing and growth suppression effect concentration was assessed to be 5 μg/mL puromycin. This concentration was used in subsequent selection experiments for positive *SV40LT*-transduced cells; Figure S3: Detection of *SV40LT* gene expression in WT TDSCs and iTDSC#6 by qPCR assay. *SV40LT* expression was only detected in early-passage iTDSC#6 (P7) and late-passage iTDSC#6 (P34) but not in WT TDSCs. N.D.: non-detectable; Figure S4: Basal protein expression of tendon-related markers in WT TDSCs and iTDSC#6. IF staining for tendon-related markers in Triton X-100 permeabilized samples of WT TDSCs and iTDSC#6 in growth medium. All tested cells were positive for SCX, TNMD and COL1 expression with similar distribution patterns. SCX and COL1 were mainly detected in cytoplasm while TNMD was detected in both nucleus and cytoplasm; Figure S5: Fabrication process of modifying 6-well TCP for gel-free tenogenesis model. Typical macroscopic view of TDSC differentiated on flat or anchored TCP showing formation of tendon-like structures in wells with anchors only; Figure S6: Tendon-like tissues generated by scaffold-free 3D tenogenesis model using WT TDSC and iTDSC#6 showed similar morphology. 3D tendon-like constructs were stained by picrosirius red. Polarized light imaging revealed strong birefringence signals, indicating that aligned collagen fibres successfully formed recapitulating structural characteristics of ECM in native tendons. Scale bar indicates 500 μm.

## Abbreviations

The following abbreviations are used in this manuscript:

TDSCs	Tendon-derived stem cells
MSC	Mesenchymal stem cell
WT	Wild type
ATCC	American Type Culture Collection
ECACC	European Collection of Authenticated Cell Cultures
JCRB	Japanese Collection of Research Bioresources
PBS	Phosphate-buffered saline
DMEM-HG	High-glucose Dulbecco’s modified Eagle’s medium
FBS	Fetal bovine serum
P/S	Penicillin–streptomycin
rcf	Relative centrifugation force
PFA	Paraformaldehyde
SV40LT	SV40 large T antigen
qPCR	Quantitative real-time polymerase chain reaction
PDMS	Polydimethylsiloxane
SCX	Scleraxis
TNMD	Tenomodulin
COL1	Type I collagen
ECM	Extracellular matrix
H_2_O_2_	Hydrogen peroxide
NAR	Nuclear aspect ratio
