# Supplementary material for: Immortalized Rat Tendon-Derived Stem Cells for Tendon Tissue Engineering
Source: Bioengineering (Basel). 2026 Mar 18;13(3):354. doi: 10.3390/bioengineering13030354 (PMC13024414; doi:10.3390/bioengineering13030354)
Supplement: Supplementary file 1 [file bioengineering-13-00354-s001.zip › bioengineering-4171871-supplementary.pdf]

Supplementary Figures

Searching of immortalized tendon-derived stem cell from various sources

Date of search: 13/6/2025

Search target: Immortalized tendon-derived stem cells or tendon-derived cell line with MSC-like properties

PRISMA flow diagram of searching of immortalized tendon-derived stem cell

Identification

Records identified from:
PubMed (n=17)
Web of Science (n=3)
Scopus (n=16)

Total n=36

→ Records removed before screening:
Duplicated: n=22

Screening

Records screen for title and abstract:

n=14

→ Records removed:
Non-related: n=12

Included

Related articles:

n=2

Related results:

1. Immortalized murine tenocyte cells: a novel and innovative tool for tendon research
2. Immortalized Tendon-Derived Cell Lines which Possess a Stem Cell-like Capacity and Form Fibrocartilaginous Tissues in Vivo

Searching of immortalized tendon-derived stem cell from non-profit cell banks

Identification & Screening

1. American Type Culture Collection (ATCC)
-> search key: "immortalized tendon stem cell"

Cell line found: 5, Tendon MSC: 0

1. European Collection of Authenticated Cell Cultures (ECACC)
-> Home>Products>Cell cultures>Browse the entire collection>
Collection Groups: Cell lines,
Cell Type: Stem cell & Stem cell/Fibroblast

Cell line found: 19, Tendon MSC: 0

2. Japanese Collection of Research Bioresources Cell Bank (JCRB Cell Bank)
Home > Cell Search > Cell Search Results

Cell line with tendon origin: 0

Included

Immortalized tenocytes with MSC phenotypes: 0

Searching of immortalized tendon-derived stem cells from commercial cell line suppliers

Identification & Screening

1. Identify cell lines directly from search engines: Google & Bing (first 20 pages of results)
Search key: [purchase order "immortalized" "tendon" "stem cell"]
Google found immortalized TDSC: 0
Bing found immortalized TDSC: 0

2. Identify cell lines from the cell line provider companies obtained from step (1.)
Commercial immortalized MSC cell line providers found from Google & Bing: 5

Immortalized TDSC found: 0

Immortalized tenocytes found: 7
a) AcceGen: 1
b) Applied Biological Materials: 1
c) InnoProt: 1
d) Cells Online: 2
e) Creative Bioarray: 2

Included

Immortalized tenocytes with MSC-like phenotypes:
➔ Applied Biological Materials: 1
Same cell line found from previous literature searching,
Ink4a/Arf knock out murine tenocyte

Figure S1. PRISMA flow diagrams of immortalized tendon-derived stem cells in academic databases, non-profit cell banks and commercial cell line suppliers. Bibliometric analysis and commodity search retrieved two immortalized cell lines displaying MSC-like phenotypes with tendon origin, including immortalized murine tenocyte and TT-D6 cell, however no rat tendon-derived stem cell line was found.

Optimizing puromycin drug concentration for *SV40LT*-transduced cell selection

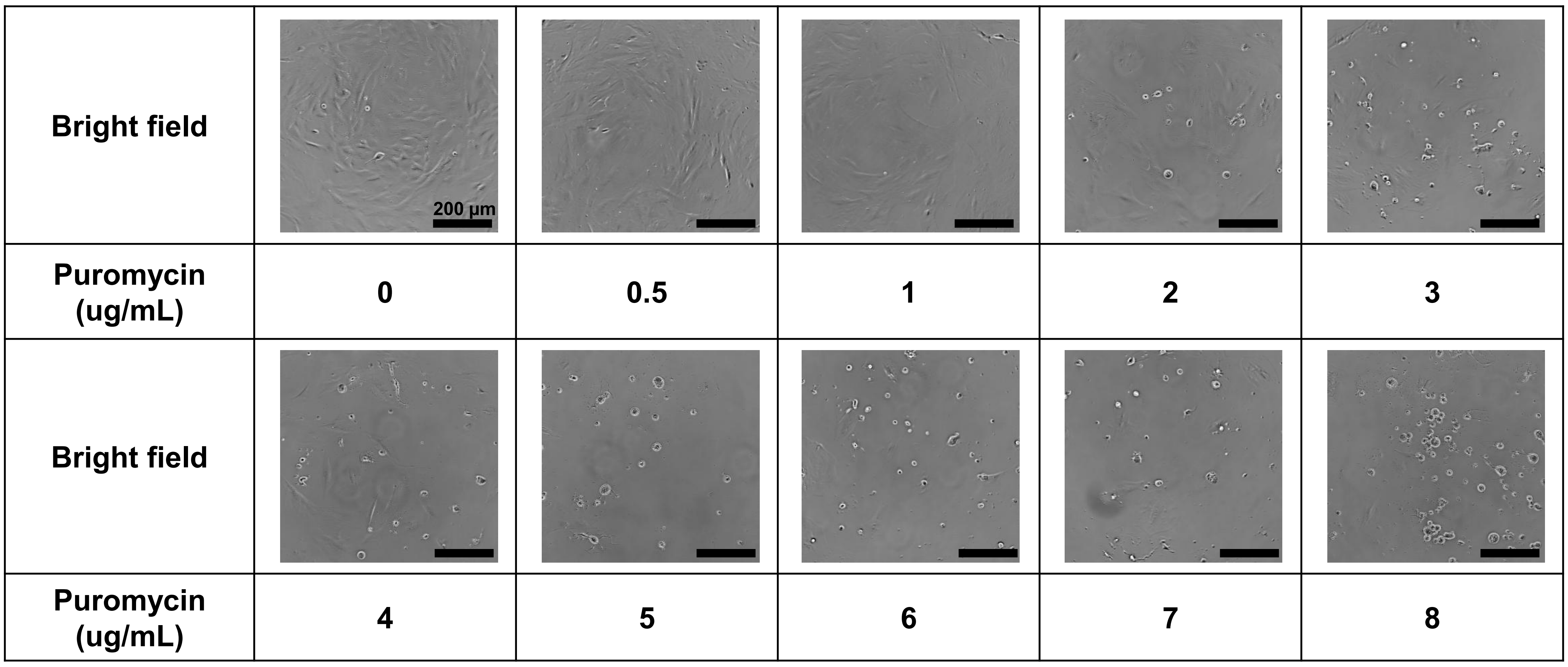

Figure S2. Puromycin resistance of WT TDSCs was determined by visual inspection of cell growth. Cells were cultured in growth medium containing up to 8 µg/mL of puromycin for 48 hours in 96 well plates. Optimal killing and growth suppression effect by puromycin was assessed to be 5 µg/mL puromycin. This concentration was used in subsequent selection experiments for positive *SV40LT*-transduced cells.

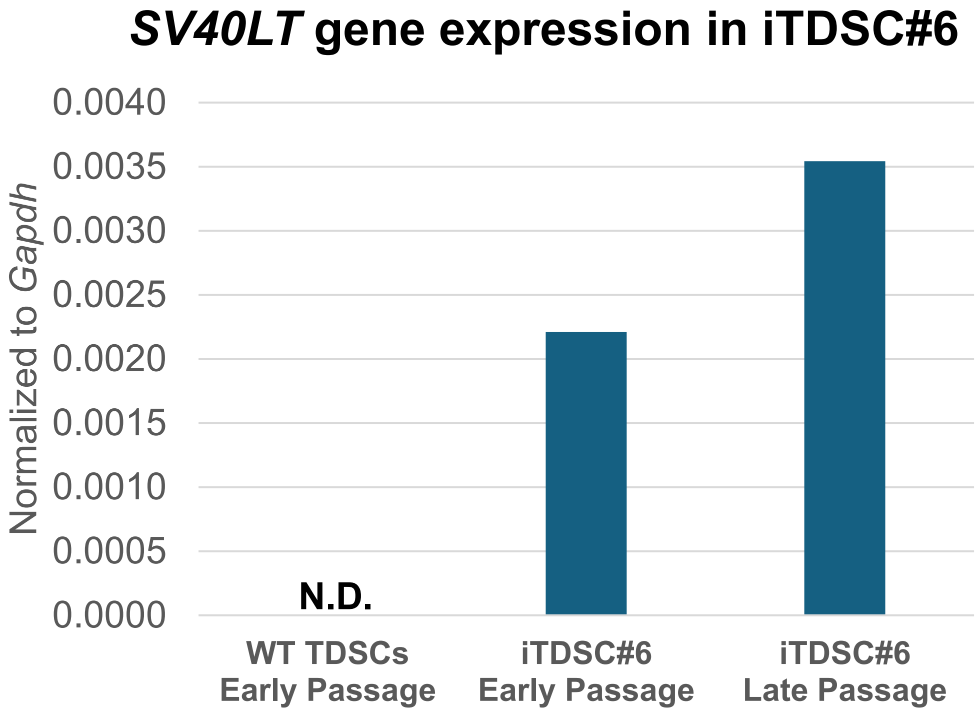

Figure S3. Detection of *SV40LT* gene expression in WT TDSCs and iTDSC#6 by qPCR assay. *SV40LT* expression was only detected in early passage iTDSC#6 (P7) and late passage iTDSC#6 (P34) but not in WT TDSCs. N.D.: Non-detectable.

Detecting basal protein expression of tendon-related markers in WT TDSCs and iTDSC#6

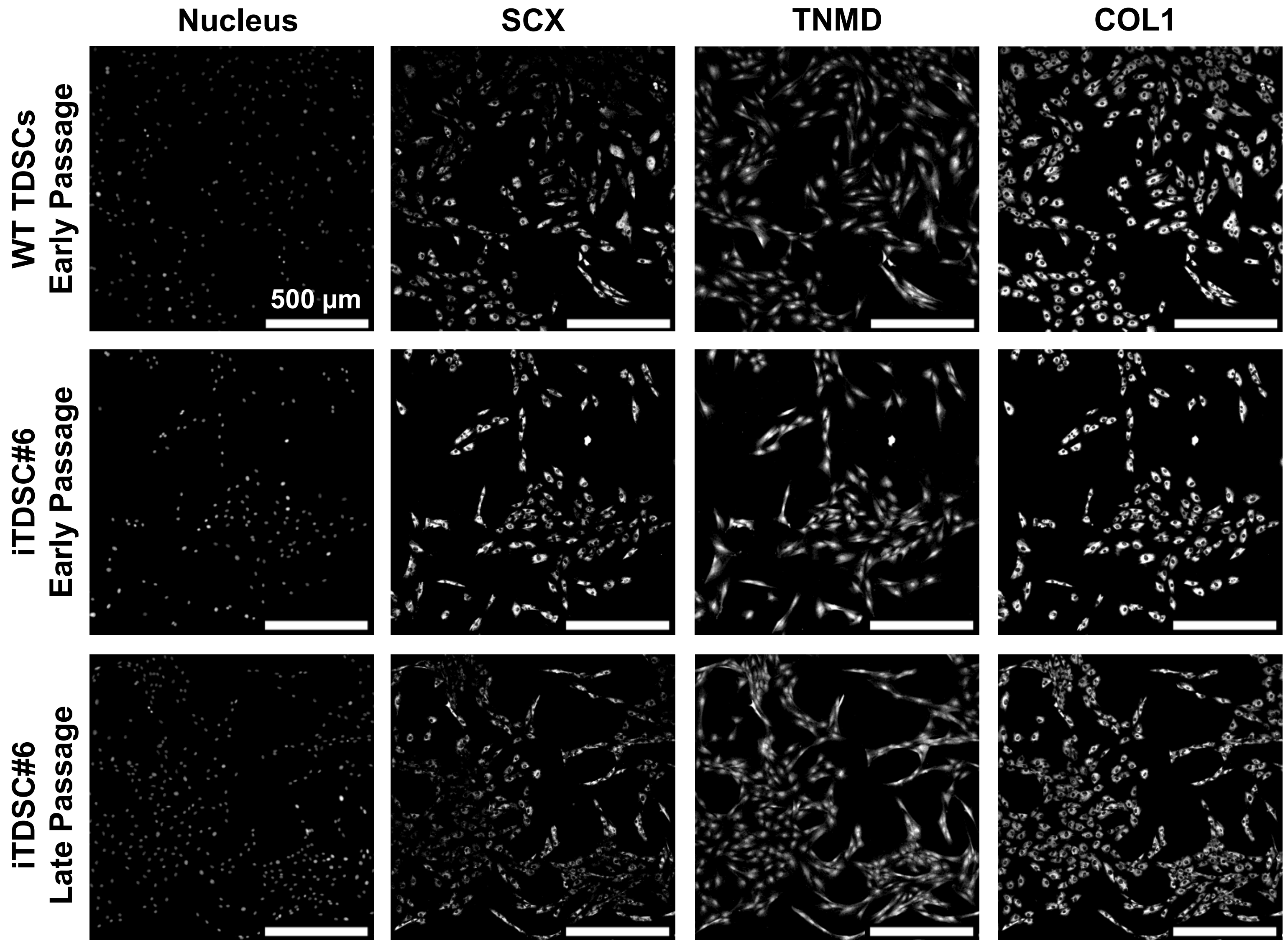

Figure S4. Basal protein expression of tendon-related markers in WT TDSCs and iTDSC#6. IF staining for tendon-related markers in Triton X-100 permeabilized samples of WT TDSCs and iTDSC#6 in growth medium. All tested cells were positive for SCX, TNMD and COL1 expression with similar distribution pattern. SCX and COL1 were mainly detected in cytoplasm while TNMD was detected in both nucleus and cytoplasm.

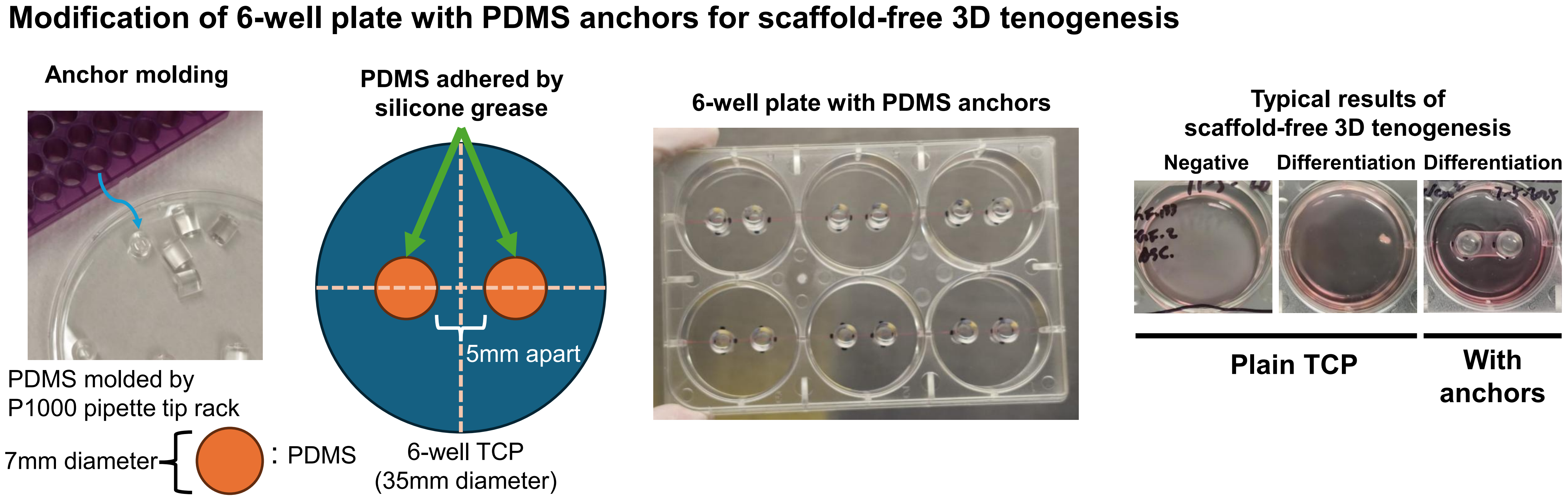

Figure S5. Fabrication process of modifying 6-well TCP for gel-free tenogenesis model. Typical macroscopic view of TDSCs differentiated on flat or anchored TCP showing formation of tendon-like structure in wells with anchors only.

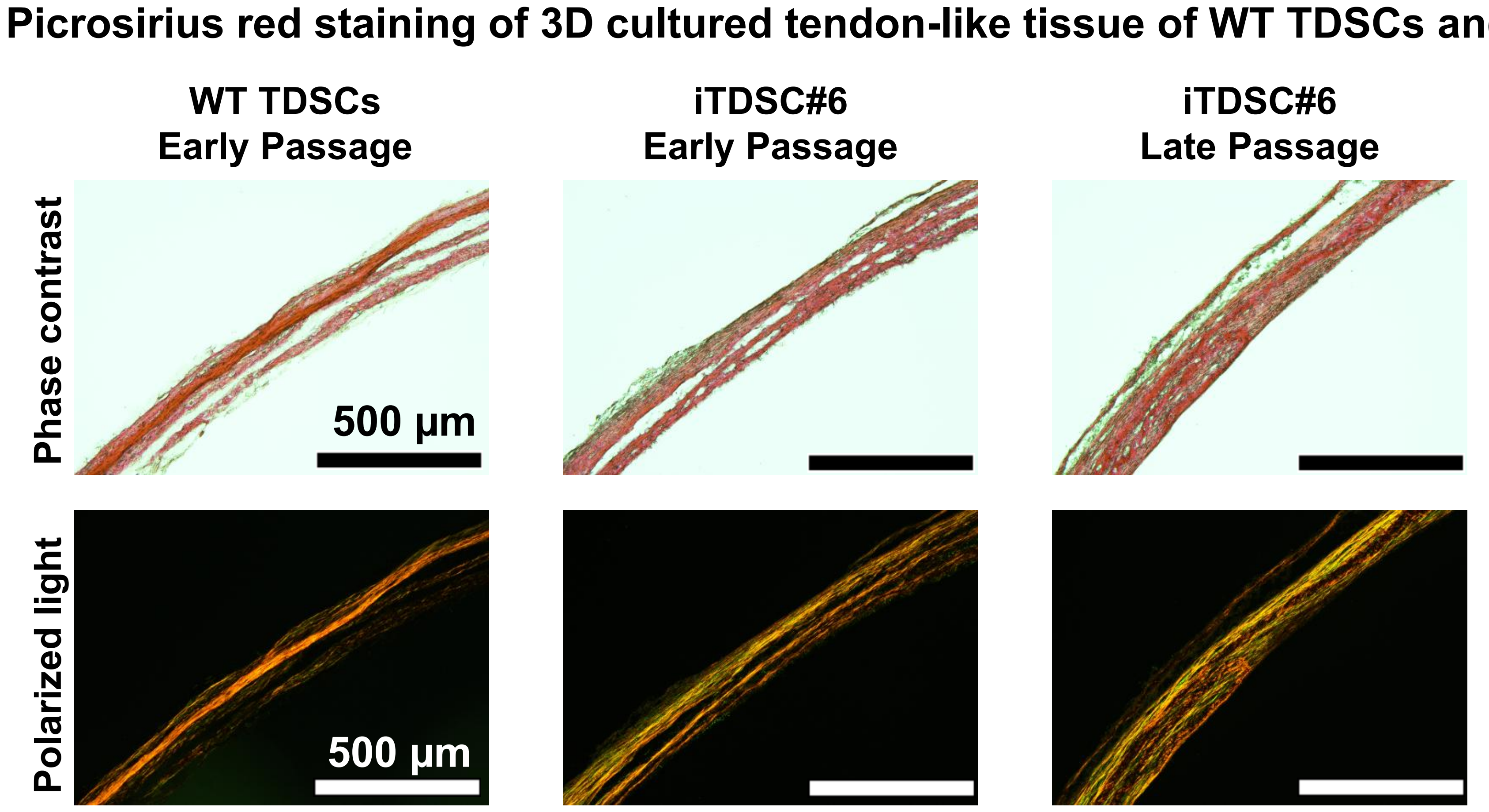

Figure S6. Tendon-like tissues generated by scaffold-free 3D tenogenesis model using WT TDSCs and iTDSC#6 showed similar morphology. 3D tendon-like constructs were stained by picrosirius red. Polarized light imaging revealed strong birefringence signals indicating aligned collagen fibres were being successfully formed recapitulating structural characteristics of ECM in native tendon. Scale bar indicates 500 µm.
